# New Perspectives of Colloids for Biological Applications

**DOI:** 10.3390/ijms26136028

**Published:** 2025-06-23

**Authors:** Agnieszka Ewa Wiącek

**Affiliations:** Department of Interfacial Phenomena, Faculty of Chemistry, Maria Curie-Skłodowska University, Maria Curie-Skłodowska Sq. 3, 20-031 Lublin, Poland; agnieszka.wiacek@mail.umcs.pl

The field of colloid systems is still a developing scientific area, but a very promising one for many practical applications. This Special Issue of the *International Journal of Molecular Sciences*, entitled New Perspectives of Colloids for Biological Applications, is dedicated to recent advances in the research of colloids and colloid-based systems, focused on highlighting recent, interesting investigations and new perspectives conducted in leading laboratories around the world. The manuscripts included in this Book focus on research data from the field of molecular chemistry; the production, modification, study, and application of colloids and colloidal systems; and the application of highly specialized methods to advance science and, above all, improve health and quality of life. This Reprint covers two main topics. The first group of the collected articles concerns application potential in medical and pharmaceutical sciences; the second covers agricultural, nutritional, and cosmetic applications. All the listed articles (12 in total) represent the latest trends in research on innovative colloidal systems for biological applications.

Aleksander Czogalla et al. contributed a paper entitled “Development of stable, maleimide-functionalized peptidoliposomes against SARS-CoV-2,” in which they presented a step-by-step process for the development of maleimide-functionalized liposomes that, when decorated with a SARS-CoV-2-binding peptide, could inhibit the progression of COVID-19 infection. A major emphasis was placed on defining the optimal lipid composition and conditions for the formation of pegylated liposomes. The authors proposed that the developed nanocarrier technology could be used as a universal platform for the construction of many antiviral agents.

Sonia Kudłacik-Kramarczyk and Marcel Krzan et al. contributed the paper “Study on the Effect of Emulsifiers on the Properties of Oleogels Based on Olive Oil Containing Lidocaine”, which reports and investigates the synthesis of olive oil-based oleogels, which are among the healthiest and most valuable vegetable fats, rich in unsaturated fatty acids and antioxidants such as vitamin E. Two types of surfactants were used: TWEEN 80, which lowers surface tension and stabilizes emulsions, and SPAN 80, which acts in oil-dominated phases. The oleogels were enriched with lidocaine, an active substance commonly used as a pain reliever and local anesthetic. This research characterized the obtained oleogels through their medical applications, paying particular attention to the influence of surfactant type and amount as well as the active substance on their physicochemical properties. The results indicated that SPAN-stabilized oleogels exhibited better stability and favorable mechanical properties, making them promising candidates for medical applications, particularly in pain relief formulations.

Izabela Nowak et al. contributed a paper entitled “Solid Lipid Nanoparticles Incorporated with Retinol and Pentapeptide-18—Optimization, Characterization, and Cosmetic Application” in which the stability of lipid nanocarriers containing retinol and peptides was investigated. To confirm the effectiveness of method optimization (high shear homogenization, HSH) and proper selection of substrates, SLN dispersions were obtained in three combinations: 1—non-incorporated SLNs; 2—SLNs containing only retinol; and 3—SLNs containing retinol and pentapeptide-18. It was confirmed that the optimized dispersion of lipid nanoparticles incorporated with retinol and oligopeptide led to the desired physicochemical parameters over the required storage period, and the possibility of using the obtained raw material as an ingredient in cosmetic products with anti-aging properties was indicated.

Cheng Peng, Li Guo, and co-workers contributed a paper entitled “Topical Delivery of Dual Loaded Nano-Transfersomes Mediated Chemo-Photodynamic Therapy against Melanoma via Inducing Cell Cycle Arrest and Apoptosis” which presents characteristics of transfersomes (TFS) designed to facilitate transdermal delivery of the chemotherapeutic drug 5-Fluorouracil (5-FU) and the PS Imperatorin (IMP) for combined chemo-photodynamic therapy for melanoma. The cytotoxic and phototoxic effects of TFS-mediated PDT (TFS-UVA) were investigated in A375 cells and mice. The authors also demonstrated that TFS-UVA generated intracellular ROS, induced G2/M phase cell cycle arrest, and promoted cell apoptosis.

Huiqiong Yan et al. contributed a paper entitled “Synthesis, Optimization and Molecular Self-Assembly Behavior of Alginate-g-Oleylamine Derivatives Based on Ugi Reaction for Hydrophobic Drug Delivery” in which, on the basis of previous research, the authors further optimized the synthesis process parameters of alginate-g-oleylamine derivatives (Ugi-FOlT). In addition, they explored the effects of different degrees of substitution (DSs) on the molecular self-assembly properties of Ugi-FOlT and its in vitro cytotoxicity and drug release behavior to achieve an optimal alginate-based oral formulation for the delivery of hydrophobic drugs. The resultant Ugi-FOlT exhibited good amphiphilic properties, and was able to self-assemble into spherical micellar aggregates in an aqueous solution and indicated good cytocompatibility. Ugi-FOlT micellar aggregates with favorable stability also showed a certain sustained and pH-responsive release behavior for the hydrophobic drug ibuprofen.

Hongmei Xia and co-workers contributed a paper entitled “Antioxidant Activity and the Therapeutic Effect of Sinomenine Hydrochloride-Loaded Liposomes-in-Hydrogel on Atopic Dermatitis”, which presents a new preparation for drug delivery. In this case, liposomes were dispersed in the colloidal hydrogel on a mesoscopic scale. The results showed that the sinomenine hydrochloride-loaded liposomes-in-hydrogel system could effectively inhibit atopic dermatitis.

Pakorn Kraisit et al. contributed a paper entitled “Fabrication and Characterization of Pectin Films Containing Solid Lipid Nanoparticles for Buccal Delivery of Fluconazole”, in which novel FZ-loaded solid lipid nanoparticles (FZ-SLNs) were developed in pectin solutions. To produce pectin films with FZ-SLNs, four formulations were selected based on the small particle size of FZ-SLNs and their suitable polydispersity index, which provided the mucoadhesive matrices. The evaluation of mechanical properties unveiled the influence of particle size variation in FZ-SLNs on the integrity of the film. The Fourier-transform infrared spectra indicated that hydrogen bonds could potentially form between the pectin-based matrix and the constituents of FZ-SLNs. In addition, the films containing FZ-SLNs provided good permeability across the porcine mucosa and antifungal properties, suggesting the potential utility of pectin films incorporating FZ-SLNs for buccal administration.

Wiącek and Furmaniuk contributed a paper entitled “Starch-Based Polysaccharide Systems with Bioactive Substances: Physicochemical and Wettability Characteristics”, which presents the physicochemical characteristics of combinations of starch with phospholipids or lysozyme, determining the effects of starch modification (surface hydrophobization or biological additives) and preparation temperature (before and after gelatinization). Surface hydrophobization of starch carried out using an *n*-alkane film does not change its bulk properties and leads to improvements in its mechanical and functional properties. The obtained specific starch-based hybrid systems, characterized in detail by switchable wettability, raises the possibility of determining the energetic state of the starch surface and understanding the strength and specificity of interactions with substances of different polarities in biological processes and their applicability for multidirectional use.

Sujka and Wiącek contributed a paper entitled “Physicochemical Characteristics of Porous Starch Obtained by Combined Physical and Enzymatic Methods, Part 1: Structure, Adsorption, and Functional Properties”, analyzing the possibility of obtaining a porous structure for native corn, potato, and pea starches using a combination of ultrasound, enzymatic digestion, and freeze-drying methods. The structure of the native and modified starches was examined using VIS spectroscopy, SEM, ATR-FTIR, and LTNA (low-temperature nitrogen adsorption). The selected properties, such as the water and oil holding capacities, least gelling concentration (LGC), and paste clarity, showed that the corn starch was the most susceptible to the combined modification methods and was therefore best suited for the production of porous starch.

Alejandro Zentella Dehesa and Ashutosh Sharma et al. contributed a paper entitled “A Comparative and Critical Analysis for In Vitro Cytotoxic Evaluation of Magneto-Crystalline Zinc Ferrite Nanoparticles Using MTT, Crystal Violet, LDH, and Apoptosis Assay”, which presents the synthesis and characteristics of zinc ferrite nanoparticles (ZFO NPs) as a promising magneto-crystalline platform for nanomedicine-based cancer theranostics. ZFO NPs synthesized using a co-precipitation method we are characterized using UV–visible spectroscopy, FTIR spectra, XRD methods, HR-TEM, XPS, and flow cytometric analysis, confirming that the NPs were more cytotoxic towards triple-negative breast cancer cells (MDA-MB-231) as compared to breast adenocarcinoma cells (MCF-7) and normal cell lines (HEK-293). ZFO can therefore be exploited for cancer therapeutics.

Pastuszak, Jurak, and co-workers contributed a paper entitled “Insight into the Mechanism of Interactions between the LL-37 Peptide and Model Membranes of *Legionella gormanii* Bacteria”, in which they presented the properties of the model membranes formed by phospholipids isolated from *L. gormanii* bacteria cultured on both non-supplemented and choline-supplemented media. The effects of the LL-37 peptide on the intermolecular interactions, packing, and ordering under monolayer compression were determined. Penetration tests at a constant surface pressure were carried out, showing that the peptide binds to the anionic bacterial membranes preferentially due to its positive charge and can penetrate into the hydrophobic tails of phospholipids, destabilizing membrane integrity. This process can entail membrane disruption and cell death. The ability to evoke such a great membrane destabilization is dependent on the share of electrostatic, hydrogen bonding, and Lifshitz–van der Waals LL-37−PL interactions. Thus, the LL-37 peptide action depends on the changes in the lipid membrane composition caused by the utilization of exogenous choline by the *L. gormanii*.

Mohammed Ghazwani et al. contributed a paper entitled “QbD-Optimized, Phospholipid-Based Elastic Nanovesicles for the Effective Delivery of 6-Gingerol: A Promising Topical Option for Pain-Related Disorders” which presents elastic nanovesicles, constructed of phospholipids optimized by 6-gingerol (6-G), a natural chemical that may alleviate osteoporosis and musculoskeletal-related pain. A 6-gingerol-loaded transfersome (6-GTF) formulation was optimized and showed more antioxidant activity than the pure 6-G suspension. The formulation was converted into a gel to improve skin retention and efficacy, which demonstrated improved skin absorption, drug release, and antioxidant activity and has the ability to treat pain-related illnesses effectively. Hence, the described study offers a possible topical treatment for conditions connected to pain.

We would like to thank all the authors who submitted manuscripts to our Special Issue for the high quality of their papers. We also express our sincere gratitude to current and future readers. We hope that our Special Issue will be a platform for dialogue between scientists working in the field of colloids and for new perspectives on colloid-based systems for various applications, especially broadly understood biological applications.

